# Concurrence of *Plasmodium falciparum dhfr *and *crt *mutations in northern Ghana

**DOI:** 10.1186/1475-2875-4-42

**Published:** 2005-09-15

**Authors:** Frank P Mockenhaupt, J Teun Bousema, Teunis A Eggelte, Stephan Ehrhardt, Rowland N Otchwemah, Robert W Sauerwein, Ulrich Bienzle

**Affiliations:** 1 Institute of Tropical Medicine, Charité – University Medicine Berlin, Spandauer Damm 130, 14050 Berlin, Germany; 2 Dept. of Medical Microbiology, Radboud University Nijmegen Medical Centre, PO Box 9101, 6500 HB Nijmegen, The Netherlands; 3 Division of Infectious Diseases, Tropical Medicine and AIDS, Academic Medical Centre, PO Box 22660, 1100 DD Amsterdam The Netherlands; 4 Bernhard-Nocht-Institute for Tropical Medicine, Bernhard-Nocht-Strasse 74, 20359 Hamburg, Germany; 5 School of Medicine and Health Sciences, University for Development Studies, PO Box TL 1350, Tamale, Northern Region, Ghana

## Abstract

**Background:**

Both chloroquine (CQ) and sulfadoxine-pyrimethamine (SP) are failing drugs in much of sub-Saharan Africa. Previous findings suggest an association between resistance to CQ and to SP *in vivo*, *in vitro*, and on the molecular level.

**Methods:**

In 126 Ghanaian children with uncomplicated malaria, associations between mutations conferring resistance in the *Plasmodium falciparum dihydrofolate reductase *(*dhfr*; SP) and *chloroquine resistance transporter *(*crt*; CQ) genes, concentrations of residual antimalarial drugs, and gametocyte carriage were examined.

**Results:**

Mutant *dhfr *alleles and the CQ-resistance allele *crt *T76 were strongly associated with each other. Isolates exhibiting the *dhfr *triple mutation seven times more likely also contained *crt *T76 parasites as compared to isolates without the *dhfr *triple variant (*P *= 0.0001). Moreover, both, isolates with the *dhfr *triple mutation (adjusted OR, 3.2 (95%CI, 1.0–10.4)) and with *crt *T76 (adjusted OR, 14.5 (1.4–150.8)) were associated with an increased likelihood of pre-treatment gametocytaemia. However, *crt *T76 did not correlate with gametocytaemia following SP treatment and no selection of *crt *T76 in SP treatment failure isolates was observed.

**Conclusion:**

These results confirm an association between CQ and SP resistance markers in isolates from northern Ghana. This could indicate accelerated development of resistance to SP if CQ resistance is already present, or vice versa. Considering the enhanced transmission potential as reflected by the increased proportion of isolates containing gametocytes when resistant parasites are present, co-resistance can be expected to spread in this area. However, the underlying mechanism leading to this constellation remains obscure.

## Background

Chloroquine (CQ) and sulfadoxine-pyrimethamine (SP) are the most frequently used antimalarial drugs in sub-Saharan Africa. With the spread and intensification of drug resistance of *Plasmodium falciparum *more effective combinatory drug regimes are now introduced in many African countries [1]. However, CQ and SP are cheap and widely available and will most likely be in use for some more years, particularly in home treatment [2-4].

In several East African countries where SP was introduced in response to intense CQ-resistance, the drug has gradually lost efficacy although the pace of this development is subject to controversy [5,6]. Previously, the core mutations linked with resistance to CQ and SP were found to be associated with each other in isolates from southern Ghana [7]. Although CQ and SP are structurally unrelated and the mutations conferring resistance are located on separate chromosomes [8-10], this finding suggests that parasites resistant to CQ may acquire resistance to SP more easily than sensitive ones, or *vice versa*. Studies in murine models [11] and *in vitro *[12,13] support this hypothesis. If this was true, the spread of CQ resistance could pave the way for an accelerated development of SP resistance, and thus, bear substantial importance to the health systems of affected regions. In addition, transmission potential may be increased in both SP and CQ resistant parasites [14,15].

Recently, mutations in the *P. falciparum dihydrofolate reductase *(*dhfr*) gene in northern Ghana were observed not only to be predictive for SP treatment failure but also to be associated with increased pre-treatment gametocyte carriage [16]. Here, associations between *dhfr *alleles, the core mutation in the *P. falciparum chloroquine resistance transporter gene *(*crt *T76), residual antimalarials, and gametocyte carriage in children with uncomplicated malaria were re-examined.

## Methods

*P. falciparum *isolates were collected from children with uncomplicated malaria participating in a treatment trial in Tamale, Northern Region, Ghana, at the end of the rainy season 2002. The results of this trial and on the *dhfr *and *dihydropteroate synthetase *gene (*dhps*) patterns are described elsewhere [16,17]. In the present report, data from all 126 children with complete follow-up after SP treatment for whom *dhfr *and *dhps *were genotyped are analysed. The study protocol was approved by the Ethics Committee, Ministry of Health, Northern Region, and by the Health Research Unit, Ministry of Health, Accra, and parents' informed consent was obtained.

Patients were children aged 6–59 months with uncomplicated *P. falciparum *monoinfection (≥ 2,000 ≤ 200,000/μL) [18,19]. None had severe malnutrition, a febrile disease other than malaria, "danger signs" [18], or severe malaria [20]. Treatment consisted of a single dose of SP (25/1.25 mg/kg, Fansidar, Roche, Switzerland), and success was monitored according to current guidelines [17,19].

Venous blood was collected into EDTA. Asexual parasites and gametocytes were counted per ≥ 200 and 500 white blood cells, respectively, on Giemsa-stained thick blood films, and densities were calculated based on a putative mean WBC count of 8,000/μL. Gametocyte counts one week following treatment were available for 104 children. Pre-treatment levels of CQ and pyrimethamine in blood were measured by ELISA assays [21] with limits of detection of 10 ng/mL and 25 ng/mL, respectively.

*P. falciparum dhfr*, *dhps*, and *crt *alleles were assessed by restriction fragment length polymorphisms (RFLP) of amplicons generated by nested PCR assays applying hot start procedures (HotStart *Taq*, Qiagen, Germany). Primers and conditions are described elsewhere [22,23] as are restriction enzymes and RFLP conditions to characterize the codons *dhfr*, 16, 51, 59, 108, 164; *dhps*, 436, 437, 540, 581, 613; and *crt*, 76. Laboratory strains were used as controls for PCR and RFLP assays. Electrophoresis was perfomed on 3% GTG agarose (FMC Bioproducts, USA) gels.

Frequencies were compared by χ^2^-test or Fisher's exact test, and continuous variables by Student's t-test, Mann-Whitney U-test, or Kruskal-Wallis test as applicable. Logistic regression models were used to adjust for potential confounders of the presence of resistance mutations and to identify risk factors for gametocytaemia.

## Results

The median age of the 68 girls and 58 boys was 28.5 months (range, 6–59). Median axillary temperature and geometric mean parasite density were 38.8°C (range, 37.5–41.0) and 36,358/μL (range, 2,068–175,333/μL), respectively. Gametocytes were found in 19% (24/126) of the children at a geometric mean density of 50/μL (16–1,120/μL). CQ in blood was observed in 66% (83/126; median, 32 ng/mL, range, 10–718) but pyrimethamine in only two children (78 & 326 ng/mL).

The *dhfr *alleles Thr-108, Val-16 and Leu-164 were not detected. Classifying mixed alleles as mutant, *dhfr *Ile-51 occurred in 56% (70/126), Arg-59 in 65% (82/126), and Asn-108 in 72% (91/126). Triple *dhfr *mutations (Ile-51/Arg-59/Asn-108) were seen in 47% (59/126), and quadruple (triple *dhfr *+ *dhps *Gly-437) and quintuple (triple *dhfr *+ *dhps *Gly-437/Glu-540) variants in 44% (55/126), and 0.8% (1/126), respectively. *Crt *T76 occurred in 80% (101/126) of the isolates.

The concomitant occurrence of *dhfr*, *dhps*, and *crt *alleles in identical isolates was analyzed. All *dhfr *mutations were linked with each other. *Dhfr *Ile-51 was associated with Asn-108 (OR, 29.8; 95%CI, 8.0–161, *P *< 0.0001) and Arg-59 (OR, 7.7; 95%CI, 3.3–19,5, *P *< 0.0001). All isolates exhibiting *dhfr *Arg-59 additionally had Asn-108 (*P *< 0.0001). Notably, in isolates with *dhfr *variant parasites, the CQ-resistance allele *crt *T76 occurred significantly more frequent than in the respective wildtype isolates (Tab. 1). Separating isolates into such with e.g. *dhfr *codon 108 pure wildtype, wildtype & mutant, and pure mutant, the prevalence of *crt *T76 was 67.5% (23/35), 82.4% (14/17), and 86.5% (64/74), respectively (χ^2^_trend _= 6.1; *P *= 0.01). Basically the same trend was seen for *dhfr *codons 51 (*P *= 0.002) and 59 (*P *= 0.002, data not shown). *Vice versa*, in isolates with *crt *wildtype, with both *crt *wildtype & T76, and *crt *T76 alone, the *dhfr *triple mutation was detected in 16% (4/25), 53% (9/17), and 55% (46/84), respectively.

**Table 1:** Associations between dhfr and crt mutations in 126 Ghanaian children with uncomplicated malaria

*dhfr *mutation	Prevalence of *crt *T76 mutation (%)	Odds ratio (95%CI) of *crt *T76 being present			
		
		Unadjusted	*P*	Adjusted for CQ in blood*	*P*
51					
absent	67.9 (38/56)	1		1	
present	90.0 (63/70)	4.3 (1.5–12.5)	0.002	5.0 (1.8–13.8)	0.002
59					
absent	63.6 (28/44)	1		1	
present	89.0 (73/82)	4.6 (1.7–13.0)	0.0007	4.4 (1.7–11.4)	0.002
108					
absent	65.7 (23/35)	1		1	
present	85.7 (78/91)	3.1 (1.2–8.6)	0.01	3.2 (1.2–8.2)	0.02
triple					
absent	68.7 (46/67)	1		1	
present	93.2 (55/59)	6.3 (1.9–26.6)	0.0006	7.0 (2.2–22.5)	0.001

*dhfr*, dihydrofolate reductase gene, *crt*, chloroquine resistance transporter gene; 95%CI, 95% confidence interval; CQ, chloroquine; *, Potential confounders were tested for in logistic regression models including age, sex, parasite density, *dhps *alleles, and axillary temperature. Adjusted odds ratios include the presence of chloroquine in blood as the only factor found to be associated (univariate odds ratio, 3.2; 95%CI, 1.2–8.6; *P *= 0.01).

The prevalence of *crt *T76 increased with increasing number of any *dhfr *mutations: it was 63% (20/32) in isolates with pure *dhfr *wildtype, 74% (26/35) in isolates with single or double mutations, and 93% (55/59) in isolates exhibiting the *dhfr *triple mutation (χ^2^_trend _= 13.1; *P *= 0.0003). Adjusting for CQ in blood, isolates with *dhfr *triple mutations revealed a seven-fold increased odds of concomitantly exhibiting *crt *T76 (Tab. 1). *Dhps *alleles neither correlated with *dhfr *alleles nor with *crt *T76 (data not shown).

*Dhfr*, *dhps*, and *crt *alleles were examined with respect to pre-treatment gametocytaemia. As reported elsewhere [16], children were significantly more likely to harbour gametocytes in the presence of *dhfr *51, 59, or triple mutations. This was also true for isolates exhibiting *crt *T76 (Tab. 2). Gametocytes were not observed in any (0/21) isolate with wildtype alleles for both *dhfr *and *crt*, but in 16% (8/50) of isolates exhibiting either *dhfr *or *crt *mutations, and in 29% (16/55) of isolates revealing both, *dhfr *triple and *crt *T76 (χ^2^_trend _= 8.7, *P *= 0.003). Adjusting for additional factors influencing gametocytaemia, i.e. high parasite density arbitrarily set as >50,000/μL, the presence of CQ in blood, and axillary temperature, both the *dhfr *triple mutation and *crt *T76 were associated with an increased likelihood of gametocytaemia (Tab. 2). *Dhfr *alleles or *crt *T76 were not associated with gametocyte density (data not shown).

**Table 2:** Associations with pre-treatment gametocytemia in 126 Ghanaian children with uncomplicated malaria

Factor	Prevalence of gametocytemia (%)	Odds ratio (95%CI) of gametocytemia			
		
		Unadjusted	*P*	Multivariate	*P*
CQ in blood					
None	27.9 (12/43)	1		1	
Present	14.5 (12/83)	0.4 (0.2–1.2)	0.07	0.3 (0.1–1.0)	0.04
Ax. temperature	n.a.	0.3 (0.1–0.6)	0.0008	0.2 (0.1–0.5)	0.0009
Parasite density					
< 50,000/μL	26.3 (20/76)	1		1	
≥ 50,000/μL	8.0 (4/50)	0.2 (0.1–0.8)	0.01	0.2 (0.1–0.8)	0.02
*dhfr *triple mutation					
Absent	10.4 (7/67)	1		1	
Present	28.8 (17/59)	3.5 (1.2–10.2)	0.009	3.2 (1.0–10.4)	0.049
*crt *76 mutation					
Absent	4.0 (1/25)	1		1	
Present	22.8 (23/101)	7.1 (1.0–304)	0.04	14.5 (1.4–150.8)	0.02

Ax., axillary; n.a., not applicable

One week after treatment, gametocytes were observed in 63% (66/104) of the children. Gametocytaemia following SP treatment was associated with pre-treatment *dhfr *mutations, e.g. *dhfr *Asn-108, OR 3.1; 95%CI, 1.3–7.6. In children harbouring *crt *T76 parasites, gametocytes were only slightly more frequent (66% (54/82)) than in patients with *crt *wildtype parasites (55% (12/22), *P *= 0.3).

Finally, selection of *crt *T76 in SP treatment failures was examined. No selection was observed: In matched pairs of isolates obtained from 35 children suffering SP treatment failure, *crt *T76 was present in 86% (30/35) of pre-treatment isolates and in 80% (28/35) of isolates obtained at treatment failure.

## Discussion

In this study on *P. falciparum *isolates from northern Ghana, two major findings are presented. First, SP and CQ resistance markers are strongly associated with each other, independent of residual antimalarials. Second, both are associated with an increased prevalence of gametocytes.

This study has several limitations and particularly the finding of an association between unrelated mutations needs caution in interpretation. Because polyclonal infections predominate in the area [24], the detection of a mutant allele in an isolate does not necessarily mean that all clones carry the mutation. Thus, it cannot be stated whether the linkage between the resistance markers is a true one, i.e. on the chromosomal level, or reflects co-occurrence in individual isolates. The limited number of *crt *wildtypes also hampered proper testing of a linkage disequilibrium. Although *dhfr *mutations were significantly more common in the presence of *crt *T76 this observation needs to be construed with caution since 80% of the isolates in this study harboured the latter variant. Also, it is impossible to comment whether the presence of *crt *T76 favours the presence of *dhfr *mutations or *vice versa *because this requires longitudinal observations. Likewise, the lack of temporal information impairs a clear statement on whether resistance mutations bring about increased gametocytogenesis. Due to methodology the resistance genotype of asexual parasites cannot be separated from that of gametocytes.

Despite these difficulties in drawing firm conclusions, several previous findings support the hypothesis of a linkage disequilibrium between mutations associated with SP resistance and CQ resistance. Early reports from the 1950s and 1960s indicated that in areas or patients with established pyrimethamine-resistance in Nigeria, Burkina Faso, and Venezuela, CQ exhibited a reduced activity (reviewed in [25]). In a murine malaria model, CQ resistance could be induced in pyrimethamine-resistant parasites but not in sensitive ones [11]. In isolates from Cameroon, the *in vitro *activity of pyrimethamine was ten times lower in CQ resistant than in sensitive *P. falciparum *[13]. Similar but less pronounced differences have also been observed in other studies [26,27]. In southern Ghana, we previously observed that the *dhfr *core mutation Asn-108 was three times more likely found in isolates exhibiting *crt *T76 than in isolates comprising *crt *wildtype parasites [7]. The reason for this apparent association between resistance to CQ and pyrimethamine or SP is obscure since both drugs have distinct modes of action and resistance to these is determined by mutations on different chromosomes [8-10]. One alluringly simple explanation could be that parasites in the study area have merely become resistant to both drugs, possibly as a result of drug pressure. In this regard, previous, simultaneous or sequential treatment with CQ and SP could have selected for resistant parasites which subsequently persisted for a longer period than the drugs can be detected in blood. However, pyrimethamine was seen in only two children which indicates that it is rarely used in this community and which argues against drug-induced selection for SP resistance. In addition, the results are corrected for the presence of CQ in blood which can be detected for approximately one month after intake [28]. This does not exclude the possibility of selected and persisting parasites but renders it rather unlikely. Alternatively, the association between *crt *T76 and *dhfr *mutations could reflect a rapid mutator phenotype with the ability of accelerated resistance to multiple drugs. This hypothesis originates from *in vitro *studies observing that parasites resistant to common antimalarials acquire resistance to structurally unrelated drugs more rapidly than susceptible strains. The genetic basis of this phenomenon is unknown but suggested to involve an increased frequency of mutations *per se *and consequently a higher probability of modified proteins which could also include drug targets [12]. In fact, selection for high-grade pyrimethamine resistance *in vitro *has been shown to enhance the degree of overall genomic polymorphism [29]. In this regard, it is noteworthy that *crt *T76 was more frequently observed with increasing number of *dhfr *mutations, i.e. with increasing degree of SP resistance.

In the present study, the association between the resistance markers meets with an increased presence of gametocytes in isolates comprising mutant *dhfr *or *crt *alleles. Gametocytaemia seemed to reflect a rather long duration of infection as can be deduced from its low prevalence in the presence of factors suggestive for acute disease, i.e. high body temperature and parasite density, and previous CQ treatment (Tab. 2). Again, an increased frequency of resistance mutations in gametocytaemic children could result from previous drug-related selection as outlined above. However, increased gametocytaemia preceding treatment has also been observed in infections subsequently found to be CQ resistant [30-32]. Hallett *et al. *[15] reported that in patients with *crt *T76 parasites, gametocyte density was highly increased one week following CQ treatment. In addition, gametocytes from patients carrying *crt *T76 parasites produced 38 times higher oocyst burdens in the mosquito as compared to *crt *wildtype parasites. In Tamale, both residual CQ levels and parasites with the *crt *T76 mutation are abundant [33], and a clear association of *crt *T76 and pre-treatment gametocyte prevalence is seen. Only one *crt *wildtype isolate contained gametocytes impairing a sound analysis of the effect of *crt *T76 on gametocyte density. However, increased gametocyte production by *crt *T76 parasites [15] in the presence of residual CQ could partially explain the present finding. The synthesis of both, *crt *and *dhfr *mutations being associated and increased gametocytaemia in their presence, gives rise to a grim scenario: Given that CQ resistant parasites have an improved transmission potential [15] the association with SP resistance would contribute to an accelerating spread of resistance to both drugs, particularly in areas where CQ resistance is frequent. In the present study, neither selection of *crt *T76 in SP treatment failure was observed nor a significantly elevated proportion of post-treatment gametocytaemia among children initially harbouring *crt *T76 parasites. Although this was not expected and would reflect an extraordinary rapid process, both observations may be influenced by the small sample size.

## Conclusion

The present data provide evidence supporting a hypothesis on a connection between resistance to CQ and SP suggesting that both, CQ and SP resistance favour transmission. This needs to be verified by carefully designed longitudinal studies in regions of differing levels of drug resistance and endemicity. *Per se*, antimalarial treatment must be effective, and more effective than CQ and SP, not only to reduce treatment failures but also the transmission of potential co-resistance. Eventually, as this has been shown to counterbalance enhanced transmission of resistant parasites [15] the results strongly support combinatory treatment including artemisinine-derivatives.

## Authors' contributions

FPM, JTB, and UB designed the study. RNO, SE and FPM were responsible for patient recruitment, clinical and parasitological examinations, and PCR assays. TAE measured drug concentrations. JTB and RWS did the gametocyte counts. FPM and JTB wrote the paper with major contributions of the other authors.
